# Beyond appearances: Complex endoscopic rescue therapy in a case of dual malignancy

**DOI:** 10.1055/a-2922-5250

**Published:** 2026-08-13

**Authors:** Andrada Seicean, Irina Dragomir, Cristina Pojoga

**Affiliations:** 1 Gastroenterology 37576 Iuliu Hațieganu University of Medicine and PharmacyCluj-NapocaRomania; 2 Gastroenterology 433709 Regional Institute of Gastroenterology and Hepatology Prof Dr Octavian FodorCluj-NapocaClujRomania; 3 Clinical Psychology and PsychotherapyBabes Bolyai UniversityCluj NapocaRomania

**Keywords:** endoscopy upper GI tract, dilation, injection, stenting, endoscopic ultrasonography, intervention EUS, pancreas

Advanced endoscopic techniques have expanded therapeutic options for patients with complex biliopancreatic malignancies, providing minimally invasive alternatives when conventional approaches fail. 1​ Furthermore, rescue endoscopic strategies play an increasingly important role in the management of complications and device-related adverse events.

We report a complex case of a 60-year-old woman with 1 year history of colonic adenocarcinoma treated with surgery and chemotherapy who was admitted with obstructive jaundice. Imaging demonstrated hilar lymphadenopathy causing biliary obstruction. Endoscopic retrograde cholangiopancreatography failed due to papillary friability and was complicated by self-limited bleeding. Endoscopic ultrasound (EUS)-guided hepaticogastrostomy (HGS) was performed, achieving effective biliary drainage ( Video 1 ).

**Video 1** Successful preservation of gastrointestinal continuity by endoscopic salvage therapy: enteral stent deployment through the impacted distal flange of the LAMS. Here the URL for the video.


**Fig. 1:**
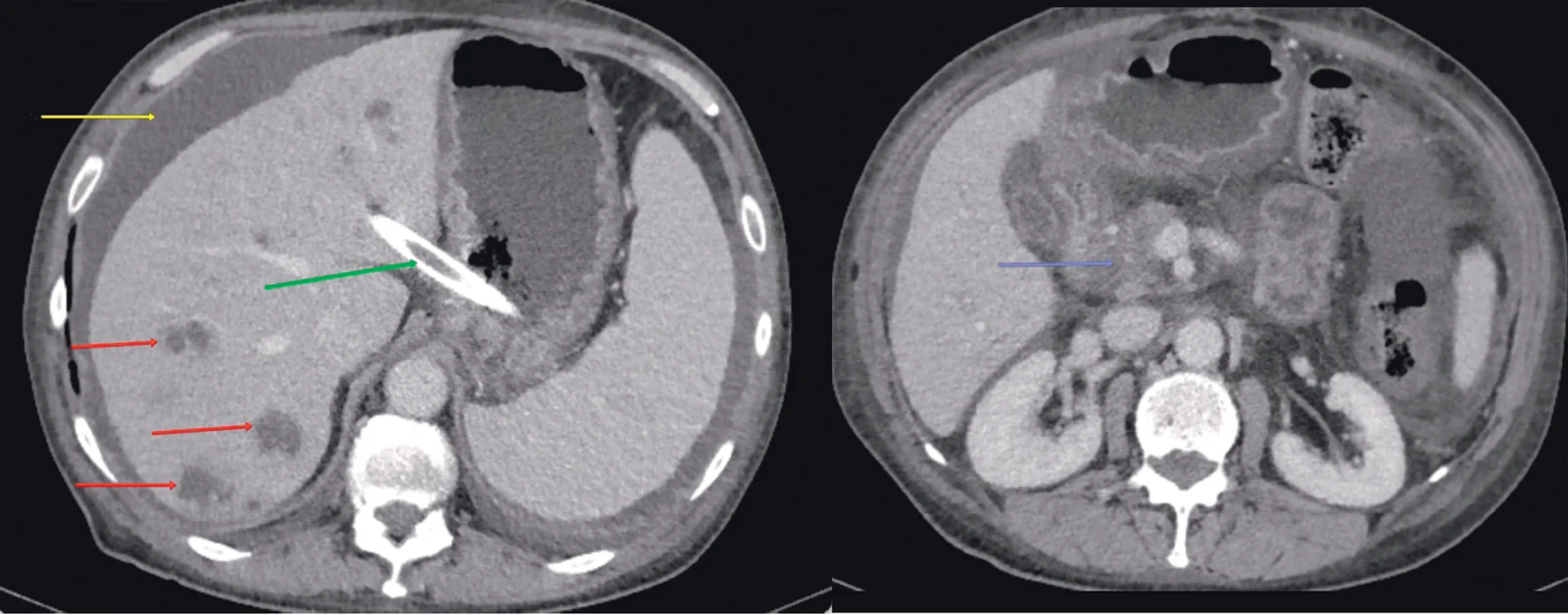
Left: CT scan showing the progression of the disease under standard chemotherapy protocol: ascites—yellow arrow; liver metastasis—red arrows; obstructed EUS-hepaticogastrostomy﻿—green arrow. Right: CT scan showing atrophy of the pancreatic head: purple arrow- pancreatic head atrophy.

Three months later, the patient developed cholangitis secondary to HGS stent occlusion by food debris. Computed tomography (CT) revealed progression of the disease by liver metastasis, ascites, gastric outlet obstruction (GOO) and atrophy of the head of the pancreas ( Fig. 1 ). EUS-guided fine-needle biopsy of hilar lymph nodes confirmed a second primary malignancy: pancreatic adenocarcinoma. Endoscopic reintervention with the exchange of the partially covered HGS stent successfully restored biliary drainage.

**Fig. 2:**
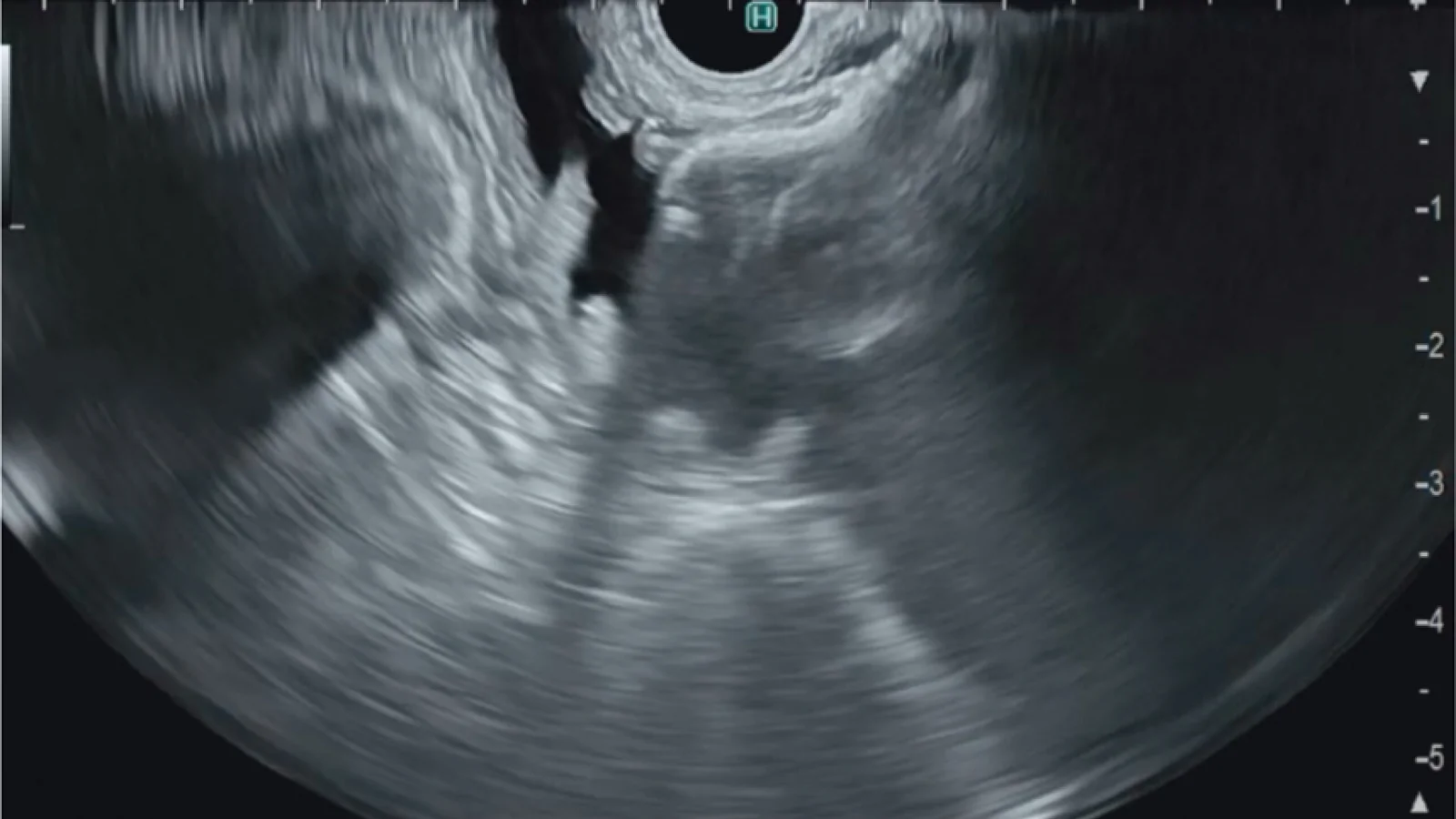
An EUS image of the distal flange of the LAMS deployed in the intestinal lumen.

**Fig. 3:**
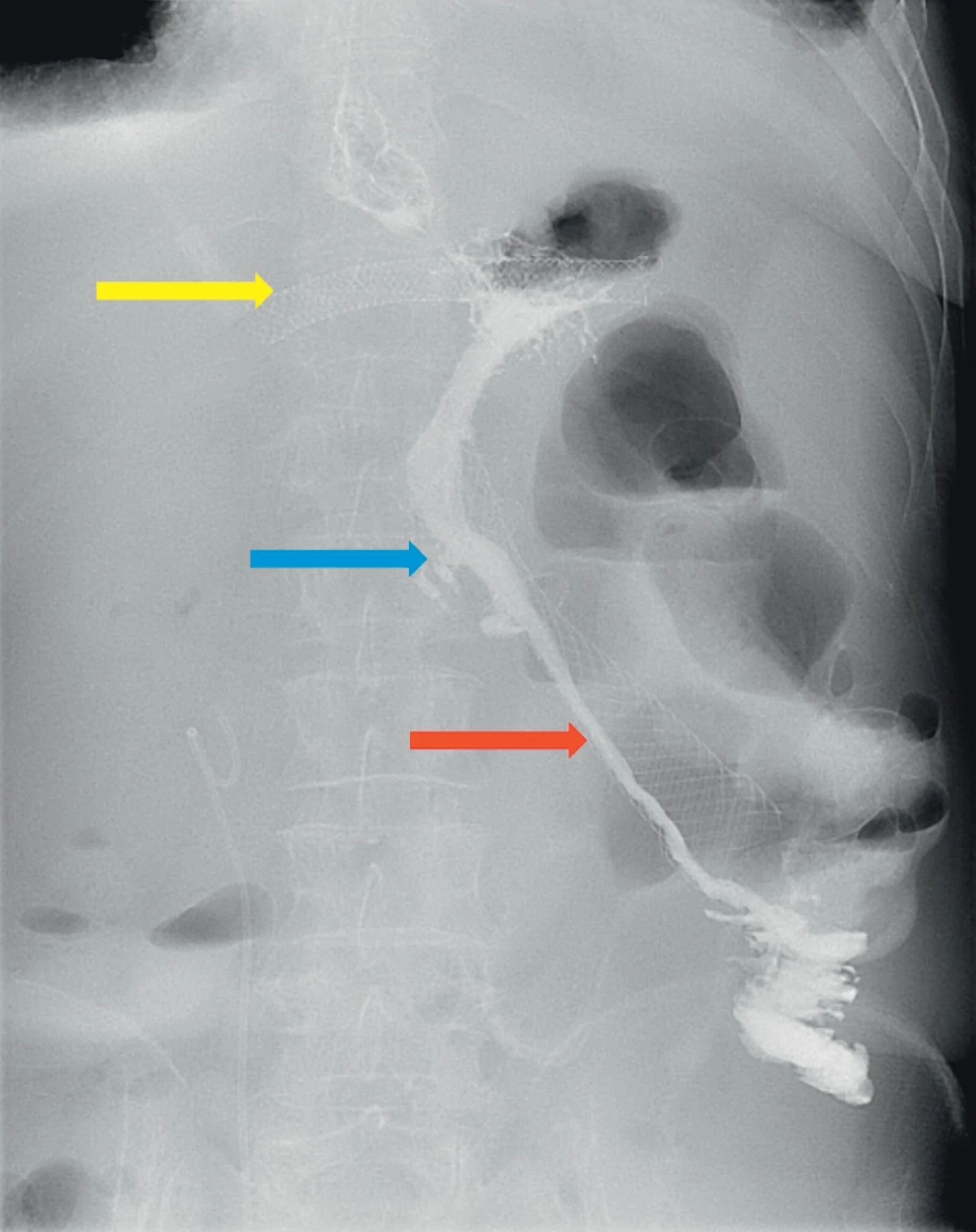
X-ray after the oral agent showing all three stents. Yellow arrow—EUS-hepaticogastrostomy, blue arrow—EUS-gastroenterostomy, and red arrow—enteral stent.

For GOO, EUS-guided gastroenterostomy using a 15-mm lumen-apposing metal stent (LAMS) was successfully performed ( Fig. 2 ). However, after 3 days of normal oral intake, recurrent vomiting developed. CT imaging demonstrated the displacement of the distal LAMS flange into the enteral wall. A complex endoscopic rescue procedure was undertaken: the enteral lumen was re-accessed under combined endoscopic and fluoroscopic guidance using a guidewire, followed by the placement of a partially covered enteral stent that re-anchored the LAMS within the bowel lumen ( Fig. 3 ).

During a 6-month follow-up, the patient remained on chemotherapy with stable disease and maintained adequate oral intake.

This case underlines the importance of salvage therapies in frail patients. EUS-guided biliary drainage and gastroenterostomy, combined with innovative salvage techniques, can successfully manage severe complications while preserving gastrointestinal continuity and quality of life.

Endoscopy_UCTN_Code_TTT_1AS_2AK
